# Psychometric evaluation of the Albanian version of the Neck Disability Index in a large cohort of patients with cervical pain

**DOI:** 10.3389/fmed.2026.1812694

**Published:** 2026-04-16

**Authors:** Elda Zeqiri, Jasemin Todri, Orges Lena

**Affiliations:** 1 Health Science PhD Program, UCAM Universidad Católica San Antonio de Murcia, Campus de los Jerónimos, N° 135 Guadalupe, Murcia, Spain; 2 ÍTEM—Innovation in Manual and Physical Therapies, Research Group, Physiotherapy Department, UCAM Universidad Católica San Antonio de Murcia, Campus de los Jerónimos, N° 135 Guadalupe, Murcia, Spain

**Keywords:** Albanian population, cervical pain, cross-cultural adaptation, Neck Disability Index, reliability, validity

## Abstract

**Background:**

The Neck Disability Index (NDI) is one of the most widely used instruments for evaluating neck pain disability. To date, no validated version has been available in Albanian. This study aimed to translate, culturally adapt, and evaluate the psychometric properties of the Albanian version of the NDI (ANDI).

**Methods:**

The ANDI was developed using a standardized forward–backward translation method. A cross-sectional study was conducted among 566 participants with neck pain. Reliability was assessed through internal consistency (Cronbach’s alpha) and test–retest reliability (Intraclass Correlation Coefficient, ICC_2,1_). Measurement error was estimated using the Standard Error of Measurement (SEM) and Minimal Detectable Change (MDC). Construct validity was examined with exploratory factor analysis (EFA) and item–total correlations (ITC).

**Results:**

The ANDI showed excellent reliability, with Cronbach’s alpha values ranging from 0.981 to 0.993 and ICC_2,1_ between 0.981 and 0.993. SEM values (0.104–0.178) and MDC values (0.288–0.493) confirmed strong measurement precision. ITC exceeded 0.96 for all items. EFA supported a unidimensional structure (KMO = 0.947, Bartlett’s test *p* < 0.001), consistent with the original instrument.

**Conclusion:**

The ANDI is a reliable, valid, and culturally adapted measure of neck disability for Albanian-speaking populations, suitable for both clinical practice and research.

## Introduction

Neck pain is one of the most prevalent musculoskeletal conditions worldwide, significantly impacting quality of life, functional ability, and productivity. It can result from various etiologies such as poor posture, cervical disc degeneration, trauma, or neurologic conditions like cervical radiculopathy. Given its multifactorial nature and its impact on daily functioning, a valid and reliable assessment tool is crucial to quantify the level of disability and monitor clinical outcomes.

Neck pain represents a substantial public health problem worldwide and is among the leading causes of years lived with disability (YLDs). According to the Global Burden of Disease Study, neck pain affects more than 200 million people globally and its prevalence continues to increase due to population aging, sedentary lifestyles, and occupational risk factors (1). In Europe, musculoskeletal disorders including neck pain are among the most common causes of functional limitation and work absenteeism. Although epidemiological data specific to Albania are limited, regional estimates from Southeastern Europe suggest that the prevalence of neck pain ranges between 20 and 30% in the adult population, particularly among individuals engaged in sedentary occupations and prolonged computer use. The increasing burden of neck pain in this region highlights the need for reliable and culturally adapted patient-reported outcome measures to assess disability and monitor treatment outcomes (2).

The Neck Disability Index (NDI), developed by Vernon and Mior in 1991, is one of the most widely used instruments for assessing self-reported disability in patients with neck pain (3). It consists of 10 items evaluating pain intensity and interference with activities of daily living such as personal care, lifting, reading, concentration, work, driving, sleeping, and recreation. Each item is scored from 0 to 5, with a total score ranging from 0 to 50, which is often converted to a percentage. Higher scores indicate greater disability. Since its development, the NDI has been extensively studied and recognized for its strong psychometric properties including internal consistency, test–retest reliability, construct validity, and responsiveness (4, 5).

NDI has been translated and culturally adapted into numerous languages and validated across diverse populations worldwide. Previous validation studies have confirmed its applicability in European, Middle Eastern, Asian, and American populations, demonstrating its widespread clinical use in assessing neck-related disability. These cross-cultural adaptations have enabled clinicians and researchers to evaluate neck pain outcomes consistently across different healthcare systems and cultural contexts. The availability of validated translations of the NDI has therefore played a critical role in facilitating international research collaboration and improving comparability of clinical outcomes across studies (6).

In addition to these adaptations, the NDI has also been validated in Brazilian Portuguese populations, demonstrating reliability ranging from reasonable to excellent and confirming adequate construct validity for assessing neck-related disability in clinical and research settings. This further illustrates the widespread international adoption of the NDI as a standardized instrument for evaluating functional limitations associated with neck pain across diverse cultural contexts (7).

Given the multifactorial nature of neck pain and its impact on daily functioning, accurate assessment of disability requires instruments that capture both physical limitations and patient-perceived functional impairment. Patient-reported outcome measures (PROMs) have become an essential component of musculoskeletal assessment because they provide direct insight into the patient’s perception of symptoms, activity limitations, and participation restrictions. Compared with clinician-based measures, PROMs are easy to administer, cost-effective, and feasible for use in both clinical practice and large-scale research settings. These instruments allow standardized evaluation of functional disability, facilitate monitoring of treatment outcomes, and support evidence-based decision-making in rehabilitation and musculoskeletal care. Consequently, PROMs such as the NDI have become widely adopted tools for evaluating neck-related disability in both clinical and research contexts (8).

Despite the wide global adoption of NDI, no validated Albanian version exists to date. This presents a significant gap in clinical practice and research in Albanian-speaking populations. Health professionals in Albania and Kosovo often resort to foreign-language instruments, which may lead to misinterpretation and reduced accuracy in evaluating patient outcomes. The lack of culturally adapted tools compromises both individual care and population-level research.

The process of cross-cultural adaptation and validation follows rigorous methodological guidelines, such as those proposed by the ISPOR Task Force (9). This includes forward and backward translation, expert committee review, pretesting with target populations, and statistical testing of psychometric properties such as internal consistency, test–retest reliability, and construct validity. The use of validated PROMs is crucial for evidence-based practice and ensures consistency in treatment planning and research (10).

The primary aim of this study is to conduct the translation, cross-cultural adaptation, and psychometric validation of the NDI for the first time in the Albanian language. By doing so, this study aims to provide clinicians and researchers with a reliable and validated tool to measure neck-related disability in Albanian-speaking patients. This is essential for improving patient assessment, ensuring standardized data collection, and facilitating international collaboration in musculoskeletal health research.

## Methods

### Study design

A multicentric cross-sectional, observational, and methodological study was conducted to adapt and validate the NDI into the Albanian language. The study followed established guidelines for cross-cultural adaptation of self-report measures and for evaluating psychometric properties (11).

### Participants

The study included adult Albanian-speaking participants with a clinical diagnosis of neck pain, recruited online as per eCOA implementation user guide for Electronic Clinical Outcomes Assessment (eCOA) developers, solution designers/architects or individuals responsible for the electronic implementation of eCOAs, as well as Mapi Research Trust’s eCOA Team which facilitated the permission to translate the Albanian NDI version. Prior to accessing the questionnaire, all participants were presented with a digital information sheet describing the purpose of the study, the voluntary nature of participation, confidentiality of responses, and the right to withdraw at any time without consequences. Participants were required to provide electronic informed consent by selecting an agreement option before proceeding to the questionnaire. Only individuals who confirmed their consent were allowed to continue and complete the survey. The study protocol was conducted in accordance with the ethical principles outlined in the Declaration of Helsinki.

Participants were eligible for inclusion if they were aged 18 years or older, were native Albanian speakers or had sufficient proficiency in the Albanian language to understand the questionnaire, reported experiencing neck pain at the time of participation, and provided electronic informed consent to participate in the study. Individuals were not included if they were younger than 18 years of age, were unable to understand the Albanian language, or declined to provide informed consent.

Several exclusion criteria were applied to ensure that the study population represented individuals with nonspecific neck pain and that participants were able to complete the questionnaire reliably. Participants were excluded if they reported a history of cervical spine surgery, diagnosed neurological disorders, malignancy affecting the spine, or previously diagnosed inflammatory rheumatic diseases involving the cervical spine, such as ankylosing spondylitis or rheumatoid arthritis with cervical involvement. These conditions were excluded to ensure that the questionnaire assessed disability related primarily to nonspecific or mechanical neck pain rather than secondary cervical pathology.

To ensure that participants were able to understand and complete the questionnaire accurately, cognitive eligibility was assessed through self-reported screening questions included at the beginning of the online survey. Participants were asked whether they had any diagnosed neurological or cognitive condition that could interfere with the comprehension of written material. Individuals reporting cognitive impairment or severe neurological disorders were excluded from participation. Additionally, questionnaires that were incomplete or contained missing responses were excluded from the final analysis.

The sample size was determined based on recommendations for validation studies (minimum of 5–10 participants per item), with a target of at least 100 participants to ensure robust statistical analysis (12).

### Ethics approval and consent to participate

The study received ethical approval from the Albanian Health and Social Ministry Ethics Committee (Protocol ID: 208/9) and from the Ethics Committee of the Catholic University of Murcia (UCAM) (Approval ID: CE012516). It was prospectively registered at ClinicalTrials.gov under the identifier NCT06833359 (https://clinicaltrials.gov/study/NCT06833359?cond=Albanian%20Neck%20Disability%20Index&viewType=Card&rank=1). All methods were performed in accordance with the relevant guidelines and regulations. Written informed consent has been obtained from the patients. Research involving human research participants must have been performed in accordance with the Declaration of Helsinki.

### Measurement

Neck Disability Index (NDI): A widely used self-report questionnaire assessing neck-specific disability across 10 items, each scored from 0 (no disability) to 5 (complete disability), for a total score ranging from 0 to 50. Higher scores indicate greater disability (13, 14).

Albanian version of the NDI (ANDI): The NDI was translated and culturally adapted into Albanian following internationally accepted procedures.

### Procedure

The NDI was translated into Albanian using a standardized forward-backward translation method. Two bilingual translators independently translated the original English version into Albanian. A synthesis version was then back translated by two native English speakers blinded to the original.

The pre-final Albanian version of the NDI was administered to a pilot group of 83 native Albanian speakers to evaluate clarity, comprehension, and cultural relevance of the translated items. Although cross-cultural adaptation guidelines typically recommend a minimum of 30 participants for pre-testing, a larger sample was intentionally included in this study to capture a broader range of demographic characteristics and to obtain more comprehensive feedback regarding linguistic clarity and item interpretation. Participants were asked to report any difficulties in understanding the items or response options, and minor wording adjustments were made based on their feedback (11).

To assess test–retest reliability, participants completed the questionnaire a second time after a 72-h interval. Although COSMIN guidelines commonly recommend an interval of 7 to 14 days between administrations, shorter intervals may be appropriate when evaluating conditions such as neck pain that may fluctuate over time. The 72-h interval was therefore selected to minimize the risk of clinical changes in symptom severity while still reducing the potential influence of immediate recall of previous responses.

### Data analysis

Data analyses were performed using Statistical Package of Social Science (SPSS) v.26, with statistical significance set at *p* < 0.05. There were no missing values. Each AODI item was described as mean and standard deviation (SD).

### Factor analysis

Exploratory factor analysis (EFA) with principal component extraction was conducted to explore the dimensionality of the ANDI. Prior to the factor extraction, sampling adequacy was assessed using the Kaiser–Meyer–Olkin (KMO) measure and Bartlett’s test of sphericity, which determine whether the data are suitable for factor analysis. A principal component analysis (PCA) with varimax rotation was employed as the extraction method to maximize interpretability of the factor loadings. The number of factors to be retained was identified based on Kaiser’s criterion (eigenvalues > 1) and examination of the scree plot, in accordance with commonly accepted practices in psychometric validation (15, 16).

### Reliability

Internal consistency was assessed using Cronbach’s alpha. Test–retest reliability was measured with the two-way mixed-model Intraclass Correlation Coefficient (ICC_2,1_) with absolute agreement and a single score. The ICC ranges from 0.00 to 1.00, with values between 0.60 and 0.80 indicating good reliability, and values above 0.80 reflecting excellent reliability (17).

An *α* value less than 0.70 indicates low consistency, a value between 0.70 and 0.80 reflects moderate consistency, and a value greater than 0.80 represents good internal consistency (18, 19).

The standard error of measurement (SEM) was computed using the square root of the mean squared error (√MSE) derived from the analysis of variance employed in calculating the intraclass correlation coefficient (ICC). The minimal detectable change (MDC) at a 95% confidence interval (CI) was determined using the formula: SEM × √2 × 1.96 (20, 21).

Additionally, both SEM and MDC were expressed as percentages relative to the sample mean, denoted as %SEM and %MDC, respectively.

Floor and Ceiling Effects was assessed by calculating the percentage of respondents scoring the minimum 0 or maximum 50 total score. Effects were considered significant if >15% of participants scored at either extreme.

## Results

A total of 566 participants were included in the study. The sample consisted predominantly of females (*n* = 400, 70.7%), while males represented 29.3% of the participants (*n* = 166). The mean age of the sample was 30.86 ± 13.51 years. The mean body weight was 68.26 ± 14.75 kg and the mean height was 168.76 ± 8.83 cm, corresponding to a mean body mass index (BMI) of 23.9 ± 4.5 kg/m^2^.

Regarding clinical characteristics, the mean neck pain intensity score was 1.67 ± 0.47. Based on pain duration, 189 participants (33.4%) reported acute neck pain (<6 weeks), while 377 participants (66.6%) reported chronic neck pain (>12 weeks).

In terms of educational background, most participants held a bachelor’s degree (65.2%), followed by a master’s degree (13.4%), while smaller proportions reported college education or doctoral degrees. The sample included individuals from a wide range of professional backgrounds, with students, physiotherapists, economists, and managers representing the most frequent occupational categories.

As per exploratory factor analysis (EFA) of ANDI Items the suitability of the dataset for factor analysis was confirmed by Kaiser-Meyer-Olkin (KMO) measure = 0.947, indicating excellent sampling adequacy. Values above 0.90 are considered superb, suggesting that the correlation patterns are compact and factor analysis is appropriate. Bartlett’s Test of Sphericity was significant (*χ*^2^ = 3686.060, df = 45, *p* < 0.001), indicating that the correlations between items are sufficiently large for factor analysis.

All 10 ANDI items loaded strongly on a single factor, supporting a unidimensional structure. Loadings ranged from 0.692 to 0.866, which are considered strong to very strong, suggesting that all items contribute meaningfully to the underlying construct (neck-related disability).

The highest-loading items were: Section 7 (Work): 0.866 Section 10 (Recreation): 0.865; Section 1 (Pain Intensity): 0.811. The lowest, yet still adequate loading: Section 3 (Lifting): 0.692 (Table 1).

**Table 1:** Participant’s characteristics.

Characteristics	Mean ± SD	*n*	%
Sex			
Female		400	70.7
Male		166	29.3
Age (years)	30.86 ± 13.51		
Weight (kg)	68.26 ± 14.75		
Height (cm)	168.76 ± 8.83		
Body Mass Index (kg/m^2^)	23.9 ± 4.5*		
Pain intensity	1.67 ± 0.47		
Neck pain duration			
Acute (<6 weeks)		189	33.4
Chronic (>12 weeks)		377	66.6
Education level			
PhD		14	2.5
Master		76	13.4
Bachelor		369	65.2
College		45	8.0
Student		62	11.0
Selected professions			
Student		171	30.2
Physiotherapist		59	10.4
Economist		52	9.2
Lawyer		33	5.8
Manager		38	6.7
Unemployed		27	4.8
Other professions		186	32.9

SD, standard deviation. *BMI calculated using the formula weight (kg)/height^2^ (m^2^).

Table 2 presents the psychometric properties of each of the 10 items of the ANDI based on pre- and post-test measurements. All items showed excellent reliability, with ICC_2,1_ ranging from 0.981 to 0.993, indicating that the instrument yields highly consistent results over time. The Standard Error of Measurement (SEM) ranged from 0.104 to 0.178, while the Minimal Detectable Change (MDC) ranged from 0.288 to 0.493. These values are low, demonstrating good sensitivity to change and minimal random error.

**Table 2:** Item’s factor loading components.

Items component		Value
Kaiser-Meyer-Olkin measure of sampling adequacy		0.947
Bartlett’s test of sphericity	Approx. chi-square	3686.060
	df	45
	Sig.	0.000
Section 7	Puna	0.866
Section 10	Rekreacioni	0.865
Section 1	Intensiteti i Dhimbjes	0.811
Section 8	Drejtimi i makinës	0.804
Section 2	Kujdesi Personal	0.797
Section 6	Përqendrimi	0.791
Section 4	Leximi	0.783
Section 9	Fjetja	0.744
Section 5	Dhimbja e kokes	0.742
Section 3	Ngritja e peshave	0.692

The Item-Total Correlations (ITC) were very high (0.963–0.987), and Cronbach’s alpha values for each item ranged from 0.981 to 0.993, confirming that the items are highly correlated and contribute consistently to the overall scale.

## Discussion

The present study evaluated the psychometric properties of the ANDI in 566 individuals with neck pain and found it to be a reliable and valid instrument for assessing neck-related disability. The results are consistent with previous cross-cultural validations of the NDI across different languages and populations.

The Croatian version of the NDI demonstrated excellent discriminative ability to differentiate between patients with acute, chronic, and no neck pain, confirming its clinical utility (22). Similarly, in Serbia, Jovicic et al. (23) validated the NDI in patients with cervical radiculopathy, finding high internal consistency and good test–retest reliability.

In Poland, Misterska et al. (24) reported strong construct validity and responsiveness of the Polish NDI version in patients with degenerative and discogenic disorders. The Greek version, validated by Trouli et al. (25), showed strong psychometric properties (Cronbach’s *α* = 0.85), and significant correlations with other pain and disability scales. The German version also demonstrated high internal consistency (*α* = 0.81) and excellent reliability (26), while the Turkish version was validated through comparison with three other disability scales, showing comparable sensitivity and specificity (27).

Asian versions of the NDI have also shown robust results. In Japan, Nakamaru et al. (28) reported high reliability (ICC = 0.91) and good construct validity of the NDI, while in Thailand, Uthaikhup et al. (29) validated the NDI and the Neck Pain and Disability Scale (NPDS), both demonstrating strong convergent validity (28, 29). The Simplified Chinese version validated by Lim et al. (30) confirmed responsiveness and reliability (Cronbach’s *α* = 0.85) in a large sample. Similarly, the Hebrew version showed good test–retest reliability (ICC = 0.89) and responsiveness in patients undergoing physical therapy (31). The Iranian version, adapted by Mousavi et al. (32), reported strong internal consistency and responsiveness in chronic neck pain patients.

In addition to general reliability and validity, some studies have highlighted the influence of psychological factors in NDI scores, particularly in chronic conditions such as whiplash.

The Brazilian Portuguese version of the NDI further confirmed the reliability and construct validity of the instrument, demonstrating reliability values ranging from reasonable to excellent depending on the domain assessed (7). Collectively, these studies highlight the strong cross-cultural applicability of the NDI and reinforce the relevance of adapting the instrument to different linguistic and sociocultural contexts.

Interestingly, the ICC values observed in the Albanian version were slightly higher than those reported in some previous validation studies. One possible explanation may be related to the characteristics of the study sample. Participants in the present study were predominantly young adults with relatively high educational attainment, which may have facilitated a clearer understanding of questionnaire items and contributed to more consistent responses between test administrations. Additionally, the relatively short interval between test and retest may have minimized the likelihood of clinical changes in symptom severity while maintaining stable self-reported responses (see Table 3).

**Table 3:** Test–retest reliability analysis of ANDI.

ANDI items	Pre-test (mean ± SD)	Post-test (mean ± SD)	ICC_2,1_ (95% CI)	SEM	MDC	Item total correlation	Cronbach’s alpha
Section 1	1.18 ± 1.095	1.19 ± 1.062	0.991 (0.990–0.993)	0.104	0.288	0.983	0.991
Section 2	0.70 ± 1.066	0.74 ± 1.051	0.988 (0.985–0.990)	0.117	0.324	0.976	0.988
Section 3	1.16 ± 1.187	1.16 ± 1.154	0.984 (0.981–0.987)	0.150	0.416	0.969	0.984
Section 4	1.37 ± 1.099	1.36 ± 1.077	0.991 (0.989–0.992)	0.104	0.289	0.982	0.991
Section 5	1.67 ± 1.276	1.64 ± 1.255	0.984 (0.981–0.987)	0.161	0.447	0.97	0.984
Section 6	1.34 ± 1.240	1.33 ± 1.217	0.991 (0.990–0.992)	0.118	0.326	0.983	0.991
Section 7	1.08 ± 1.134	1.06 ± 1.090	0.982 (0.979–0.985)	0.152	0.422	0.966	0.982
Section 8	1.18 ± 1.290	1.20 ± 1.252	0.981 (0.978–0.984)	0.178	0.493	0.963	0.981
Section 9	1.25 ± 1.297	1.24 ± 1.279	0.993 (0.992–0.994)	0.109	0.301	0.987	0.993
Section 10	1.13 ± 1.160	1.12 ± 1.138	0.989 (0.987–0.991)	0.122	0.337	0.979	0.989

ANDI, Albanian Neck Disability Index; ICC, intraclass correlation coefficient; CI, confidence interval; SEM, standard error of measurement; MDC, minimal detectable change.

Another important aspect to consider is the demographic profile of the study participants. In the present study, women represented approximately 70% of the sample. This sex distribution is consistent with epidemiological evidence indicating that neck pain is more frequently reported among women than men (1, 2). Similar sex distributions have also been reported in several previous NDI validation studies, including those conducted in Brazil, Germany, and Japan, where female participants constituted the majority of respondents (7, 26, 28).

The relatively high educational level observed in the current sample may also have influenced the psychometric performance of the questionnaire. Individuals with higher educational attainment may demonstrate greater health literacy and better comprehension of self-reported outcome measures, potentially contributing to the high internal consistency observed in the present study. Comparable demographic profiles have been reported in other cross-cultural validation studies of patient-reported outcome measures, where participants with higher educational backgrounds often demonstrate greater response reliability.

Moreover, the wide variety of professional backgrounds represented in the sample supports the generalizability of the findings across different occupational groups. Since neck pain is frequently associated with occupational factors such as prolonged sitting, repetitive movements, and ergonomic conditions, including participants from diverse professional environments strengthens the ecological validity of the instrument.

Overall, the demographic and professional diversity of the sample, combined with the strong psychometric performance of the instrument, supports the applicability of the Albanian version of the NDI as a reliable tool for assessing neck-related disability among Albanian-speaking populations.

Although the sample demonstrated a relatively homogeneous distribution in terms of educational level, this characteristic can be explained by the recruitment strategy used in the present study. Participants were recruited through online platforms and academic networks, which may have led to a higher participation rate among individuals with higher educational attainment. Nevertheless, the sample included participants from a wide range of professions and age groups, suggesting that the instrument was tested across diverse occupational contexts. This diversity supports the applicability of the ANDI in different segments of the population.

The ANDI demonstrated excellent test–retest reliability, with Cronbach’s Alpha value ranging from 0.932 to 0.926 in both baseline and second administration. These results are comparable to findings from other validated versions (33). For instance, the Dutch version by Jorritsma et al. (34) reported a Cronbach’s Alpha of 0.83, while the Gujarati version showed similar reliability with Cronbach’s Alpha value of 0.93 (34, 35). The Norwegian version (36) also demonstrated good to excellent internal consistency with Cronbach’s alpha 0.83 to 0.91 for the first and second administration though slightly lower than what was observed in our Albanian sample (33, 36).

In our study, ANDI demonstrated low Standard Error of Measurement (SEM) values ranging from 0.104 to 0.178 and Minimal Detectable Change (MDC) values between 0.288 and 0.493. These figures indicate minimal measurement error and strong sensitivity to change, underscoring the instrument’s precision and reliability. Comparatively, in the study by Young et al. (2019), which assessed the psychometric properties of the NDI in patients with mechanical neck pain without upper extremity symptoms, the reported MDC for the NDI was 6.9 points on a 50-point scale. This higher MDC suggests a greater threshold is needed to detect a true change beyond measurement error in their population (37).

The notably lower SEM and MDC values in our study suggest that the ANDI is more sensitive to detecting clinically meaningful changes in neck disability among Albanian-speaking patients. This enhanced sensitivity may be attributed to factors such as the short time of second index administration, rigorous translation and cultural adaptation process, the diverse participant sample encompassing both acute and chronic neck pain cases, and the standardized administration procedures employed.

These findings position the ANDI as a precise and responsive tool for assessing neck-related disability, offering clinicians and researchers a reliable instrument for both individual patient monitoring and broader epidemiological studies within the Albanian-speaking population.

Internal consistency was also excellent in our study, with Cronbach’s alpha values ranging from 0.981 to 0.993. These values exceed the commonly accepted threshold of 0.70 for research use and align with other validation studies. The Serbian version (23) reported an alpha of 0.91, while the Greek (25) version reported alphas of 0.85. Our results reinforce the strong internal coherence of the NDI items across languages and cultural contexts.

Exploratory factor analysis of the ANDI supported a unidimensional structure, with all ten items loading strongly (≥0.692) on a single factor and a KMO value of 0.947, indicating excellent sampling adequacy (33). Similar unidimensional solutions have been found in several previous studies, including the Polish version (24) and the Norwegian version (36), reinforcing the conceptual consistency of the NDI across different linguistic settings. In our study, the items related to work, recreation, and pain intensity showed the highest factor loadings and item-total correlations, suggesting these domains are most affected by neck disability in the Albanian population. This pattern mirrors findings in other populations, where functional limitations related to work and recreation have shown to be among the most sensitive indicators of neck-related disability (35, 37, 38).

Although this study provides strong evidence for the reliability and validity of the ANDI, certain limitations should be acknowledged. The study relied on self-reported data, which may be influenced by individual perceptions or social desirability bias. While the test–retest design was appropriate for reliability estimation, responsiveness to clinical change over time was not evaluated in a longitudinal framework.

Despite these limitations, this study represents the first comprehensive validation of the NDI in the Albanian language, filling a critical gap in the assessment tools available for neck pain in Albanian-speaking populations. With a large and diverse sample size (*n* = 566), this research offers robust evidence for the psychometric soundness of the ANDI, including high test–retest reliability (ICC > 0.98), excellent internal consistency (Cronbach’s alpha > 0.98), and a clear unidimensional factor structure. These findings support the use of the ANDI in both clinical practice and research, enabling accurate evaluation of neck disability and facilitating international comparisons across validated NDI versions. The rigorous methodology and broad participation enhance the generalizability and relevance of this tool in the Albanian healthcare context.

## Conclusion

Overall, the ANDI demonstrates psychometric properties that are equal to or better than those reported in previous cross-cultural adaptations. Its excellent reliability, internal consistency, and unidimensional structure confirm its suitability for use in clinical and research settings to assess neck disability among Albanian-speaking populations.

These findings suggest that the ANDI retains the conceptual and measurement integrity of the original instrument, while being culturally and linguistically adapted to the Albanian population. Therefore, the results support the conclusion that the ANDI is a psychometrically sound tool for evaluating neck disability and is ready for use in both clinical practice and future research across Albanian-speaking populations.

## Data Availability

The raw data supporting the conclusions of this article will be made available by authors under a reasonable request.
