# Correction: Psychometric evaluation of the Albanian version of the Neck Disability Index in a large cohort of patients with cervical pain

**DOI:** 10.3389/fmed.2026.1862787

**Published:** 2026-05-06

**Authors:** Elda Zeqiri, Jasemin Todri, Orges Lena

**Affiliations:** 1 Health Science PhD Program, UCAM Universidad Católica San Antonio de Murcia, Campus de los Jerónimos, N° 135 Guadalupe, Murcia, Spain; 2 ÍTEM—Innovation in Manual and Physical Therapies, Research Group, Physiotherapy Department, UCAM Universidad Católica San Antonio de Murcia, Campus de los Jerónimos, N° 135 Guadalupe, Murcia, Spain

**Keywords:** Albanian population, cervical pain, cross-cultural adaptation, Neck Disability Index, reliability, validity

In the published article, **Tables 1, 2, and 3** were in the wrong order. The correct order is:

Table 1. Participant's characteristics.

Table 2. Item's factor loading components.

Table 3. Test–retest reliability analysis of ANDI.

The original version of this article has been updated.

